# Engineering Smart Targeting Nanovesicles and Their Combination with Hydrogels for Controlled Drug Delivery

**DOI:** 10.3390/pharmaceutics12090849

**Published:** 2020-09-07

**Authors:** Kamil Elkhoury, Polen Koçak, Alex Kang, Elmira Arab-Tehrany, Jennifer Ellis Ward, Su Ryon Shin

**Affiliations:** 1 Division of Engineering in Medicine, Department of Medicine, Brigham and Women’s Hospital, Harvard Medical School, Cambridge, MA 02139, USA; kamil.elkhoury@univ-lorraine.fr (K.E.); polen.kocak@std.yeditepe.edu.tr (P.K.); kangbf@bc.edu (A.K.); 2 LIBio, University of Lorraine, F-54000 Nancy, France; elmira.arab-tehrany@univ-lorraine.fr; 3 Department of Genetics and Bioengineering, Faculty of Engineering and Architecture, Yeditepe University, TR-34755 Istanbul, Turkey; 4 Division of Genetics, Department of Medicine, Brigham and Women’s Hospital, Harvard Medical School, Boston, MA 02115, USA; jward@bwh.harvard.edu

**Keywords:** liposomes, exosomes, targeting nanovesicles, hydrogel, controlled drug delivery

## Abstract

Smart engineered and naturally derived nanovesicles, capable of targeting specific tissues and cells and delivering bioactive molecules and drugs into them, are becoming important drug delivery systems. Liposomes stand out among different types of self-assembled nanovesicles, because of their amphiphilicity and non-toxic nature. By modifying their surfaces, liposomes can become stimulus-responsive, releasing their cargo on demand. Recently, the recognized role of exosomes in cell-cell communication and their ability to diffuse through tissues to find target cells have led to an increase in their usage as smart delivery systems. Moreover, engineering “smarter” delivery systems can be done by creating hybrid exosome-liposome nanocarriers via membrane fusion. These systems can be loaded in naturally derived hydrogels to achieve sustained and controlled drug delivery. Here, the focus is on evaluating the smart behavior of liposomes and exosomes, the fabrication of hybrid exosome-liposome nanovesicles, and the controlled delivery and routes of administration of a hydrogel matrix for drug delivery systems.

## 1. Introduction

Today, one of the key challenges in bioengineering and nanomedicine is how to formulate biomaterials and nanoparticles that selectively deliver encapsulated therapeutics to specific cells or tissues, when the enhanced permeability and retention (EPR) effect is inefficient. Liposomes have been studied and investigated for more than five decades and have become a well-established drug delivery vesicle, resulting in the marketing authorization of many clinically approved liposome-based products to treat different diseases [1]. Liposomes’ resemblance to biomembranes enables superior biocompatibility and safety over other polymeric and metal-based nanoparticles, as well as the ability to deliver lipid-soluble and water-soluble molecules at the same time [2,3]. However, liposomes require surface modification with ligands to acquire smart targeting capabilities. On the other hand, some natural nanovesicles, such as exosomes, already possess these targeting capabilities. The smart behavior is granted to exosomes by the donor cells in the form of cellular and lipid adhesion molecules expressed on their surfaces that allow them to target specific types of receptor cells [4]. Furthermore, since exosomes are produced by the cells, they offer an even higher level of biocompatibility and a lower immunogenicity than liposomes, which increases their stability in systemic circulation and enhances their uptake profile and therapeutic efficacy in vitro and in vivo [5,6]. However, exosomes have limitations in terms of efficient and reproducible loading with drugs or bioactive agents. To address this issue, while equipping liposomes with smart tissue and cell targeting behavior, many research groups have created hybrid liposome-exosome delivery systems [7,8,9,10].

**Figure 1:**
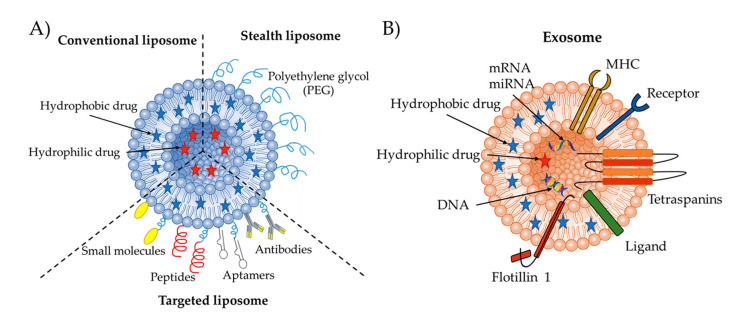
Schematic illustration of (**A**) conventional, PEGylated/stealth, and ligand-targeted liposome, and of (**B**) exosome structures.

In fact, exosomes and liposomes have many similarities (Figure 1), as both of them are nanovesicles composed of one lipid bilayer, ranging in sizes from 40 nm to 120 nm. Due to these similarities, artificial or synthetic exosome-mimetic nanovesicles are normally derived from liposomes [5]. However, liposomal and exosomal nanovesicles have major differences as well, with the main one being the complex surface composition of exosomes. The lipid composition and membrane proteins of exosomes differentiate them from other nanovesicles. Their unique lipid composition dictates their in vivo fate as they play an important role in specific interactions with serum proteins. Their membrane proteins (i.e., tetraspanins) facilitate their cellular uptake and increase the efficiency of their targeting ability. Compared to synthetic nanovesicles (micelles, liposomes and polymeric nanoparticles), exosomes are less cytotoxic, more biocompatible, can evade phagocytosis, and have an extended blood half-life [11,12,13]. Recently, head-to-head comparisons between liposomes and exosomes have been questioned because of the poor selection of controls [14]. However, all these comparisons have shown that the advantages of exosomes are the disadvantages of liposomes and vice-versa. Therefore, as mentioned before, combining these two nanovesicle types into one hybrid nanovesicle will preserve the beneficial features of both of these complimentary systems and allow for the engineering of an enhanced drug delivery targeting system.

The most common way of administering drug-loaded liposomes and exosomes is via injection. However, it is not a very effective method because it is difficult for the nanovesicles to be retained at the targeted site, and thus rapid clearance is the only inevitable outcome. One possible solution to avoid multiple injections and to release the drug over a long periods of time is to embed nanovesicles in a hydrogel system. Hydrogels have been commonly used as drug delivery matrices, as, in addition to the protection they provide to the encapsulated drugs or nanovesicles, they are able to form a drug depot following their administration at the targeted defected site and control the release rate of both nanovesicles and drugs in a time dependent manner [15,16,17,18,19,20]. Both natural and synthetic biodegradable hydrogel systems have been used for the development of these depot-forming controlled release systems. However, the main advantages of naturally derived hydrogels used as extracellular matrices (ECMs) mimicking systems are their biocompatibility, biodegradability, and promotion of cell adhesion, growth, proliferation, differentiation, and natural ECM secretion [21]. As a result, natural hydrogels are usually the preferred choice when choosing a drug delivery system. Many of the hydrogel limitations, such as low tunability and low mechanical properties, could be overcome via the synergistic effect of the incorporated nanovesicles [21,22,23]. Furthermore, the ability of drugs and nanovesicles of different sizes to be loaded and released from hydrogel systems allows for delivery via administration routes other than injection or oral. This will allow broader biomedical usages for the embedded nanovesicles, such as wound healing, bone and spinal cord regeneration, and direct cell reprogramming.

Here, we provide a comprehensive insight for liposomes, exosomes, and their hybrid nanovesicles with recent improvements in their formulation as drug delivery nanovesicles. The fabrication of hybrid nanovesicles from membrane fusion will also be highlighted. In addition, natural hydrogels used as controlled delivery systems and their usual routes of administration will be outlined.

## 2. Liposomes as Drug Delivery Vesicles

Liposomes were first discovered in the 1960s when the British Dr. Bangham noticed that phospholipids formed a closed bilayer upon contact with water [24,25]. Phospholipids are amphiphilic molecules, which, when surrounded in an aqueous medium, the hydrophobic acyl chains drive the thermodynamically favorable formation of a lipid sphere [26,27]. This formation is enhanced by electrostatic interactions, such as van der Waals forces and hydrogen bonding [28,29]. The liposomal vesicle is made up of an aqueous core encircled by a lipid bilayer and is able to encapsulate both hydrophobic and hydrophilic bioactive molecules [30,31]. Hydrophobic molecules are entrapped in the lipid bilayer with a higher efficiency than the entrapment of hydrophilic molecules in the aqueous core, due to the lower volume of hydration in the liposome core [26]. Based on their surface characteristics, liposomes can be categorized as conventional PEGylated/stealth liposomes, or ligand-targeted (Figure 1). Clinically approved liposome-based products cover 6 main therapeutic areas [1]:

- Cancer therapy: DaunoXome^®^ (non-PEGylated), Depocyt^®^ (non-PEGylated), Doxil^®^ (PEGylated), Marqibo^®^ (non-PEGylated), Mepact^®^ (non-PEGylated), Myocet^®^ (non-PEGylated), Onivyde™ (PEGylated).
- Fungal diseases: Abelcet^®^ (non-PEGylated), Ambisome^®^ (non-PEGylated), Amphotec^®^ (non-PEGylated).
- Analgesics: DepoDur™ (non-PEGylated), Exparel^®^ (non-PEGylated).
- Photodynamic therapy: Visudyne^®^ (non-PEGylated).
- Viral vaccines: Epaxal^®^ (non-PEGylated), Inflexal^®^ V (non-PEGylated).
- Rare genetic disease treatment: ONPATTRO^®^/Patisiran (non-PEGylated).

### 2.1. Conventional Liposomes

Liposomes can be formed from naturally occurring lipids that are extracted and purified, or from commercially available synthetic lipids. Conventional liposomes can be classified according to their size and lamellarity. They can be small (~100 nm) or large (~1000 nm) vesicles and can be composed of a single (unilamellar) bilayer or multiple (multilamellar) bilayers. The number of bilayers and the size of liposomes affect their encapsulation efficiency, drug release profile, physical stability upon storage, and cell internalization [32,33]. The size of liposomes and the number of bilayers is controlled via the chosen method of preparation. Multilamellar vesicles can be formed by the thin-film hydration method, large unilamellar vesicles can be produced by the freeze-thaw method, and small unilamellar vesicles can be generated with sonication or multiple extrusions through a polycarbonate membrane. Liposomes are widely used as drug delivery vesicles mainly because they are biocompatible and can increase the bioavailability while reducing the toxicity of encapsulated drugs, but also because their surface properties, charge, and size can be simply engineered to deliver their cargo into cells via adsorption onto the cell membrane, fusion with the cell membrane, micropinocytosis, or endocytosis [34].

However, the surface of the conventional liposome is usually impaired through opsonization by physical interactions with specific circulating proteins in blood. The opsonizing proteins include fibronectin, laminin, type I collagen, C-reactive protein, immunoglobulins, and complementary proteins. Though opsonization is an important natural process and is crucial for the immune response to clear dangerous pathogens, it hinders the ability of liposomes to circulate in the blood pool for a prolonged period [35]. Opsonized liposomes are recognized and cleared by the mononuclear phagocytic system (MPS) or reticuloendothelial system (RES), which are located in the liver and spleen. Another limitation of conventional liposomes is their tendency to release their cargo during circulation. To avoid this problem and to increase the circulation time of a liposomes, a hydrophilic polymer called polyethylene glycol (PEG) can be added to their surface, to create what is known as PEGylated or stealth liposomes [36,37].

### 2.2. Stealth Liposomes

The term “stealth” used in biomedical research is derived from the “low observable technology” applied to military tactics, which mainly refers to invisible nanovesicles that can avoid clearance from the bloodstream [38]. The development of long-circulating liposomes is crucial to avoid clearance by the organs of the MPS and to achieve prolonged persistence and targeted delivery of drugs. This invisibility can be achieved by decorating the outer liposome surface with stealth polymeric substances, such as PEG [39,40].

Polymeric materials, whether natural or synthetic, should be biocompatible to reduce the amount of interaction between the liposome surface and the opsonizing proteins to circumvent an immune response. PEGylated liposomes are heavier than conventional liposomes and are thus eliminated from the body by a different mechanism. This increased weight helps them to avoid enzymatic degradation and clearance via glomerular filtration [41,42,43]. The weight of PEGylated liposomes governs their clearance fate, as the heavy ones with weights above 20 kDa are primarily eradicated by the liver, whereas the lighter ones are eliminated through renal filtration [43]. PEGylated liposomes alter the pharmacokinetic profile of encapsulated drugs and thus decrease their toxicity and increase their therapeutic index. Doxil^®^, a typical PEGylated liposome encapsulating the chemotherapy drug doxorubicin, was the first nanodrug approved by the Food and Drug Administration (FDA) in 1995 [44]. Encapsulated doxorubicin in PEGylated liposomes maintained a presence in human circulation for more than 350 h and achieved a human circulation half-life time of around 90 h [45,46].

When they accumulate in the body, PEGylated liposomes mainly accumulate in tumor tissues rather than in normal tissues, thus creating a local drug depot in their accumulation area. This depot increases the drug tissue concentration and promotes a higher therapeutic effect. However, due to the EPR effect, a concentration of PEGylated liposomes in a targeted area is possible, but the efficient release of drugs is not guaranteed, even after endocytosis by the cells, as the PEG coating can sometimes constrain the drugs’ endosomal escape [47]. Moreover, a homogeneous distribution of liposomes in the targeted area is hard to achieve, especially in complex microenvironments, which can hinder sufficient treatment. Thus, active targeting drug delivery with improved strategies are required to promote efficient treatment.

### 2.3. Targeted Liposomes

Through membrane fusion or endocytosis, liposomes can deliver drugs inside the cell membrane, as both membranes are composed of phospholipids. Therefore, active targeting liposomes that enter targeted cells via receptor-mediated endocytosis should be engineered to achieve an efficient cell-specific uptake. Conjugating the appropriate targeting ligands, such as small molecules, aptamers, monoclonal antibodies, and peptides, on the surface of liposomes can modulate the cell-type-specific uptake and tissue distribution of PEGylated liposomes. The overexpression levels of the corresponding receptors or proteins on the cell surface, which these targeting ligands are bound to, influence the cellular uptake efficiency [48].

To improve cell targeting specificity, the liposomal surface can be functionalized with small molecules which possess a high binding affinity to receptors present on the cell surface. Many cancer cells overexpress folate receptors, which makes the small molecule folate a great candidate to direct the delivery of liposomes containing cancer therapeutics towards cancer cells [49,50,51]. The overexpression of sigma receptors in many cancer cell lines has paved the way for another small molecule ligand possessing a high binding affinity to these receptors: anisamide [52,53,54]. Banerjee et al., attached the anisamide moiety to liposomes and included a PEG spacer between them to improve the ligand targetability and stability, and to increase the circulation half-life [55]. This was the first study to use anisamide to target and deliver doxorubicin encapsulated in liposomes to prostate cancer cells overexpressing sigma receptors.

Aptamers are RNA or DNA sequences which exhibit high affinities and specificities towards specific cells and tissues [56]. Aptamers’ target specificities are adopted thanks to their unique three-dimensional structures. Baek et al., inserted RNA aptamer-conjugated micelles into liposomes loaded with doxorubicin to target LNCaP prostate epithelial cells expressing the prostate specific membrane antigen (PSMA), thus minimizing the systemic toxicity and side effects of the anticancer drug [57].

Receptor-specific cell-targeting and nonspecific cell-penetrating peptides (CPP) are the two peptide categories used for liposome surface functionalization [58]. When compared to nontargeted liposomes, peptide-targeted liposomes showed superior therapeutic efficacy, which was caused by the enhanced cellular uptake in target cells [59,60]. The conjugation of peptides to liposomes can be achieved through thioester linkages, sulfanyl bonds, disulfide bonds, peptide bonds, and maleimide linkages [36,61]. Ding et al., constructed cell-penetrating peptides (CPP)-modified, pH-sensitive PEGylated liposomes that displayed improved targeting and cellular internalization efficiencies on MCF-7 cancer cells [62].

The surface functionalization of liposomes via covalent coupling to the modified PEG termini distal with monoclonal antibodies (mAbs) or their fragments, such as fragment antigen-binding (Fab’) and single-chain variable fragment (scFv), can generate immunoliposomes with reduced side effects and the ability to target cells which overexpress the antigens to these antibodies [63]. Immunoliposomes have been extensively studied for cancer therapy, however, they can also be used to treat many other diseases, such as autoimmune and degenerative diseases, inflammatory and cardiovascular diseases, and infectious pathologies. Various methods have been reported for coupling antibodies to the PEGylated liposome surface, with the most common ones involving the conjugation between the PEG chains’ distal ends and the antibodies [64]. The chronic neurodegenerative disease, Alzheimer’s disease, is caused by the accumulation of neurofibrillary tangles and amyloid plaques (Aβ), two core pathological hallmarks, in the brain. Ordóñez-Gutiérrez et al., functionalized the surface of PEGylated liposomes by using a monoclonal anti-Aβ antibody to capture Aβ in the periphery and showed that these immunoliposomes had a higher therapeutic efficacy than the free monoclonal antibody [65].

## 3. Exosomes as Drug Delivery Vesicles

Cell to cell communication is important for the integrity of organisms and for maintaining tissue homeostasis. In fact, these cell communication mechanisms mostly require the coordination of signaling molecules and receptors [66]. Recently, cell to cell communication mediated via nanovesicles, mostly exosomes, has become popular due to the ability to shuttle various bioactive molecules between producing and target cells [2]. The first term of “exosome” was described 50 years ago as cellular garbage released via shedding of the plasma membrane [67]. According to the literature, exosomes can be released from almost every cell type, including lymphocytes, mesenchymal stem cells (MSC), cancer cells, epithelial and endothelial cells and dendritic cells [68,69,70,71,72,73]. Studies indicate that extracellular vesicles contain receptors involved in antigen presentation, including class I and II MHC molecules, co-stimulatory molecules such as CD83 and CD40, exosomes derived from B and T cells, and mast production [74].

Depending on the originating cell or organism, the exosome’s contents may vary, but generally, all exosomes encompass nucleic acid molecules (mRNAs, functional microRNAs, and non-coding RNAs), proteins, small molecule metabolites and lipids [75]. Additionally, the exosome’s surface contains receptors (HSP70), which are valuable for transporting materials to recipient cells and for identifying exosomes [76]. There are numerous methods to isolate exosomes, such as ultracentrifugation, differential centrifugation, chromatography, two phase aqueous systems named as polymer-based precipitation, filtration, and immunological separation. The development of a gold standard universal method that is efficient, with a high yield, but without compromising biologic function, is an active research goal [77].

Besides their ability to communicate between cells due to their small nano-metric size (∽30–150 nm [78]), exosomes are found in both the nucleus and in the cytoplasm and are also involved in the RNA processing of cells [79]. Exosomes differ from other extracellular vesicles with their unique biogenesis pathways, lipid compositions, and cargo that they can carry [76]. These vesicles, which can be obtained from all bodily fluids, have been demonstrated to have an important role in many biological functions such as intercellular communication, signal transmission, genetic material transfers and regulation of the immune response.

### 3.1. Biogenesis of Exosomes

The secretion of exosomes is mediated by multivesicular bodies (MVBs). The formation of exosomes via the MVBs pathway is eventuated by the endosomal membrane’s inward budding into the endosomal lumen. Later, the MVBs deliver their endosomal cargo to the lysosomes for degradation. Other than delivering cargo to lysosomes, these vesicles play a role in molecule secretion via plasma membrane fusion [80]. After the membrane fusion, exosomes that are found in the MVBs are dispatched into the extracellular space and then are received by a recipient cell either by plasma membrane fusion, receptor ligand binding, or endocytosis [81].

Intraluminal vesicle formation necessitates the endosomal sorting complex, which is needed for the transport (ESCRT) functions [82]. These mechanisms are composed of four different ESCRT proteins (0 to III), which cooperate to aid MVB formation, the budding of the vesicles, and protein cargo classification and sorting [83,84]. ESCRT dependent exosome biogenesis is initiated by the identification and sequestration of ubiquitinated proteins into the endosomal membranes’ particular units via ESCRT-0 binding subunits. Afterwards, the exosome will cooperate with ESCRT I-II, and will be combined with ESCRT-III, which plays a role in supporting the total complex of the budding process. Finally, after separating the buds and forming ILVs, the MVB membrane and the ESCRT-III complex will also be separated with the separation protein Vps4s’ energy [82]. Studies have mentioned that exosome biogenesis is related to an ESCRT regulation mechanism, and different ESCRT compartments and ubiquitin proteins have already been investigated in exosomes obtained from different types of cells. In addition, it has been reported that the exosomal protein, Alix, associated with several ESCRT mechanism proteins such as TSG101 and CHMP4, participates in sorting exosome cargo and membrane budding through sydnecan interactions [85]. These studies have led to a hypothesis that implies ESCRT mechanisms play a large role in exosome biogenesis.

### 3.2. Molecular Composition of Exosomes

Exosome composition may vary from cell to cell, an indication that the contents of an exosome are not only a mirror of the donor cell, but also a reflection of the sorting process [86]. Exosome cargo is comprised of various proteins, nucleic acids such as DNA, mRNA, miRNA, small molecules and lipids, which are found both inside and on the surfaces of exosomes [87,88]. A proteomic analysis of exosomes has demonstrated that some proteins originate from the cell or tissue of origin, and some proteins are common among all exosomes [85]. Typically, exosomes contain proteins with different functions, for example: tetraspanins (CD9 CD81, CD63 and CD82) involved in cell penetration, invasion, and fusion; heat shock proteins such as HSP70 and HSP90, which are involved in the stress response, which is also related to antigen binding and delivery; MVB formation proteins (Alix, TSG101) found in exosome secretion; and proteins responsible for membrane transplantation and fusion (Annexin and Rab) [89]. Among these proteins, some of which participate in exosome biogenesis like Alix, fotilin, and TSG101, are secreted upon plasma membrane spillage, while others are specifically found in exosomes and can be used as an exosome marker proteins, such as HSP70, TSG101, CD63 and CD81 [89].

### 3.3. Exosomes and Signaling

Previously, exosomes were believed to be cellular garbage with mediocre lysosomal degradation capacity. However, studies showed that exosomes were involved in various physiological processes, their functions in vivo continued to be explained, and now they are recognized as very significant for cell-to-cell communication and cellular signaling.

It is known that there are several different exosome-based mechanisms in cell-cell communication. The first is that the proteins in the exosome membrane activate intracellular signaling by interacting with receptors on target or receptor cells. Another mechanism is that the membrane proteins of exosomes can be cut by soluble fragments and proteases and can thus act as soluble ligands that bind to the receptors of the cell surface. Finally, exosomes can be engulfed by target cells and can release their cargo molecules to trigger downstream events in the recipient cells [90].

The secretion of exosomes by many different cells such as epithelial cells, stem cells, hematopoietic cells, cancer cells, and neural cells has shown that these nanovesicles can be effective in cellular physiology and pathology. Exosomes play a role in maintaining normal homeostasis, and may exert both a protective or detrimental role in human pathologies, such as cardiovascular diseases [91]. MicroRNAs are short non-coding RNAs which regulate gene expression and are enriched in exosomes, and alterations in their levels are associated with cardiovascular diseases. The cells of the heart, such as cardiomyocytes, fibroblasts and endothelial cells, secrete exosomes in response to injuries, and mediate paracrine crosstalk through microRNA levels between cardiac cell types in conditions such as cardiomyocyte hypertrophy [92,93]. In the immune system, exosomes are known to play a significant role in regulating signals by intervening innate and adaptive immune responses. Notably, there is some evidence that exosomes play a role in the spread of antigens or MHC-peptide complexes.

In addition, according to proteomic studies, exosomes have been shown to contain proteins located in cellular signaling pathways. The effects of these proteins on targeting and cellular signaling have not yet been fully disclosed, but sheds light on new studies. In particular studies, the Wnt signaling pathway, which is also known as the signal transduction pathway and plays important roles in embryo development, tissue regeneration, and cancer metastasis, has attracted attention. However, the mechanisms by which Wnt proteins can target cells are mostly unknown. Indeed, membrane bound palmitoylated Wnt proteins are not likely to be released into the extracellular space as soluble proteins. All in all, recent studies suggest that the packaging of exosomes and the release of their cargo may be promising for the downregulation of cellular signaling pathway activity [94,95,96]. Exosomes have potential as both a therapeutic target and may serve as biomarkers of disease.

## 4. Engineering Hybrid Exosome-Liposome Systems

Recent studies have revived the usage of exosomes for targeted drug delivery, with surface modifications or by producing hybrid synthetic nanovesicles. Exosomes are nanosized particles that have great potential to increase anticancer responses and targeted drug delivery. Exosomes modified by genetic or non-genetic methods can increase the cytotoxicity and targeting ability of therapeutic agents, thereby improving their effectiveness for the drug delivery [5].

**Figure 2:**
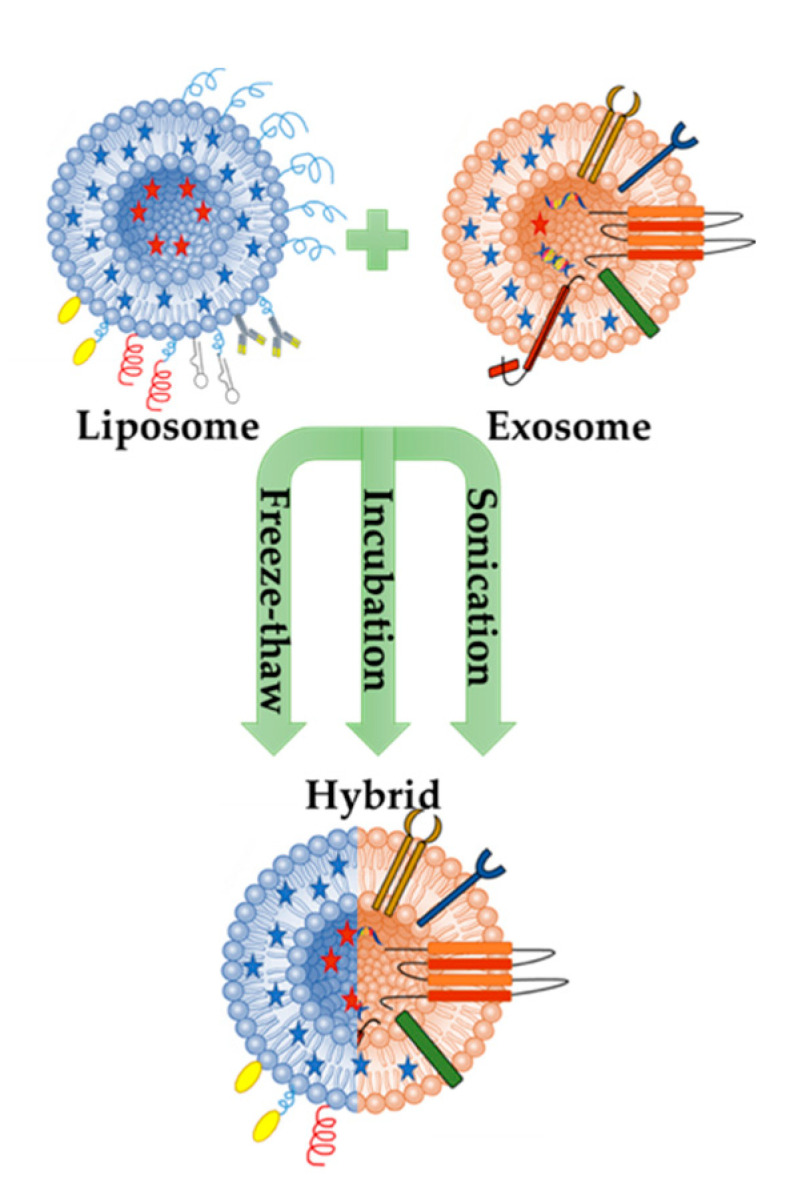
Schematic illustration of hybrid exosome-liposome nanovesicles formed by three main methods: sonication, incubation, and freeze-thaw cycles.

As mentioned briefly above, exosomes can transmit signal molecules such as miRNA, mRNA, proteins and lipids [69]. Due to their small sizes, they have the ability to escape phagocytosis and can carry and deliver the cargo in circulation. Exosomes can also pass through the blood brain barrier and placental barrier [71]. Because of their high drug delivery potential, studies have focused on the engineering of exosomes using both surface modification and hybridization with synthetic nanocarriers, such as liposomes (Figure 2) [9].

Therefore, to increase the delivery efficiency of exosomes, Sato et al. tried to form an exosome-liposome hybrid fusion using the freeze-thaw method. The aim of this study was to modify the exosome surface to reduce the immunogenicity of the exosome and also increase the colloidal stability. The result of the study demonstrated a new way to hybridize exosomes into a biological nanocarrier, which could be used to transport exogenous hydrophobic lipids, as well as hydrophilic cargos to recipient cells via membrane fusion method [7].

According to the literature, the exosome’s lack of size turnability could be disadvantageous for the encapsulation of bioactive molecules with various sizes. Current evidence for drug delivery is mostly related to micro RNAs and siRNAs, or particles with a smaller size than cas9 expressing plasmids. Therefore, new strategies should be developed for increasing the efficacy of both encapsulation and targeting for drug delivery. In one study, the successful delivery of the CRISPR-Cas9 system in MSCs was achieved via hybrid exosomes produced through simple incubation with liposomes [9].

Exosomal membrane engineering, in other words, modifying exosomes through membrane fusion with synthetic liposomes, aims to make exosome liposome hybrids to increase the half-life of exosomes in blood. In addition, due to the hydrophobic properties of lipid molecules, lipids have been shown to prevent the direct loading of exosomes. It is not easy to make genetic changes in the exosome lipid membrane because there is more than one protein in the lipid biosynthesis process, and the process of separating the lipid from the parent cell to exosome has not been clearly demonstrated.

Therefore, in recent studies, new strategies have been proposed for the preparation of hybrid particles designed by the fusion of the exosomal and liposomal membranes via freeze-thaw cycles [97]. The fabrication of these hybrid particles is one of the strategies used to abstain possible safety problems associated with the usage of allogenic nanovesicles, and to avoid the inefficient isolation yield or the long time required to produce and isolate exosomes. In addition, studies have focused on the development of optimized microfluidic based approaches, and ready-to-use GMP compatible equipment is available to expand production.

Although current studies have shown that it is possible to determine the exosomal lipidic and protein content using lipidomic and proteomics tools, the issue of whether these methods will lead to the production of efficient targeting liposomes in vivo is still being explored. Indeed, extracellular vesicles have been known to have targeting potential for some types of cells over the past five years, but in most cases, they have failed to show the expected therapeutic results following systemic administration. Subsequent unsuccessful trials have revealed some shortcomings in the methods utilizing exosomes as targeted drug delivery nanovesicles. Now, the main prerequisites for using nanovesicles to deliver and target specific drugs are: (i) efficient loading with a drug/molecule to elicit a therapeutic effect; (ii) good stability during circulation in the bloodstream before achieving therapeutic goals (preservation of size, structure and drug load); (iii) the ability to block the uptake of macrophages and the capability of traveling for a long time to reach their cellular targets and cargos; and (iv) being nontoxic, nonimmunogenic, and biocompatible. Because of the many similarities between liposomes and exosomes (as noted in Section 2 above), both nanovesicles have been used as hybrid molecules to improve targeted drug delivery.

All in all, studies on exosomes, nano-sized vesicles encapsulating proteins, and nucleic acids have grown in number over the past years due to their important roles in cell-cell communication. While the composition and biogenesis of mammalian-derived exosomes have been the focus of several studies, others have demonstrated the usage of these vesicles both as diagnostic and therapeutic tools for the drug delivery. In addition, the biocompatible properties of exosomes and liposomes with appropriate modifications can increase the cellular targeting efficiency as a drug delivery system. One of the main focuses of this review is to summarize examples of exosome and liposome modifications, and the delivery of therapeutic molecules, as well as passive and active loading approaches.

## 5. Nanovesicles-Hydrogels Interactions

Hydrogels are mainly noted for their composition and ability to maintain a stable structure. As a result of these desired properties, hydrogels have been extensively studied as engineerable ECM mimics for tissue engineering and drug delivery applications [98]. Natural proteins or polysaccharides, such as collagen, alginate, chitosan, gelatin, or hyaluronic acid (HA), can be used to form hydrogels [99]. Natural hydrogels are better suited for drug delivery applications compared to nanovesicles, mainly because of their formulation stabilities and drug administration routes. For example, liposome-based technology presents several shortcomings such as instability, rapid clearance from blood circulation, capture by the reticuloendothelial system, and rapid degradation [100]. To combat this, encapsulating nanovesicles in hydrogels can protect them from rapid clearance and can enhance their membrane integrity and mechanical stability. Additionally, hydrogels’ physical, mechanical, and biological properties can be improved and tuned by the incorporated nanovesicles [21]. Other properties such as charge, pore size, hydrophobicity, and hydrophilicity can be also be tuned by nanofunctionalization with nanovesicles to form controlled release composite hydrogel delivery systems that have been used for many biomedical applications (Table 1).

### 5.1. Liposome-Loaded Hydrogels

Gelatin is a natural protein that is produced by denaturing collagen. Due to its favorable biodegradability, biocompatibility, and low antigenicity, gelatin is mostly used in biomedical and pharmaceutical applications. However, rapid degradation and a low mechanical modulus are two main limitations for using unmodified gelatin in biomedical applications. To surpass these limitations, gelatin is usually chemically modified into gelatin methacryloyl (GelMA) by the addition of methacrylate groups to the amine-containing side groups [116]. In the presence of a photoinitiator, this methacrylation reaction allows for the light polymerization of gelatin into a hydrogel. Undamaged cell adhesive arginine-glycine-aspartic acid (RGD) motifs and matrix metalloproteinase degradable amino acid sequences help in retaining the excellent biocompatibility and bioactivity of gelatin by the fabricated GelMA hydrogels.

**Figure 3:**
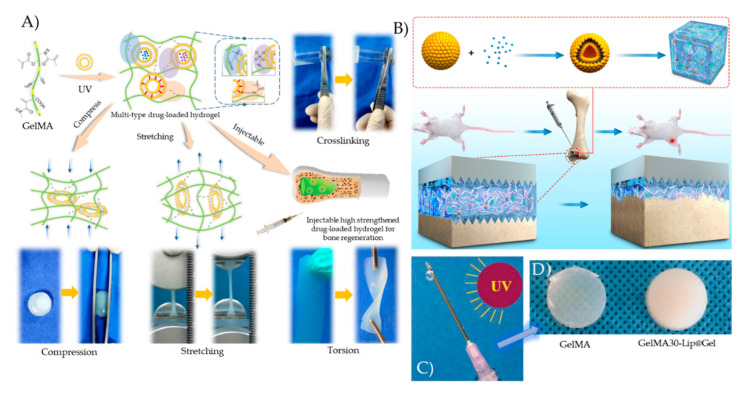
(**A**) Liposome-GelMA hydrogel with controlled release of bone regeneration drugs and enhanced mechanical properties. Reproduced with permission from [15], Elsevier, 2018. (**B**) The bone regeneration mechanism promoted by Melatonin-loaded liposomes embedded in a GelMA-Dopamine hydrogel. Reproduced from [102], Hindawi, 2020. (**C**) The mechanism of UV induced crosslinking and (**D**) the appearance of UV crosslinked GelMA and Gemcitabine-loaded liposomes embedded in GelMA (GEM30-Lip@Gel). Reproduced from [101], Taylor & Francis, 2018.

Although GelMA is biocompatible and can be used for depot drug delivery, its big pores cannot control the release of drugs and often leads to a burst release. To solve this issue, many groups have embedded liposomes loaded with bioactive molecules in the GelMA matrix. In addition to offering a controlled release, the liposome integration improves the GelMA’s mechanical properties due to the hydrogen bonding that forms between the GelMA polymer chains and the phospholipid bilayers. Cheng et al., reported that such a mechanically enhanced liposome-GelMA hydrogel can sustain stretching, torsion, and compression, and studied the controlled release of deferoxamine, a hydrophilic drug, from this composite hydrogel (Figure 3A) [15]. 80% of deferoxamine was released from the GelMA hydrogel in the first 4 h compared to about 25% released from the liposome-GelMA hydrogel. The controlled release of the composite hydrogel led to a significant promotion of angiogenesis and osteogenic differentiation in vitro and in vivo, influencing the adhesion or proliferation of MC_3_T_3_-E_1_ and HUVECs cells.

In a more recent study, Xiao et al., generated a sustained Melatonin (MT) release system composed of MT liposomes embedded in a GelMA-Dopamine (DOPA) hydrogel, and studied its release behavior and ability to induce implant osseointegration in an osteoporotic state (Figure 3B) [102]. As for the release behavior, the samples exhibited various release characteristics depending on the density of the hydrogel network, with 5% GelMA constructs having only 5 days of sustained release and 20% GelMA constructs exhibiting up to 25 days of sustained release. The developed system could be used for the treatment of implant loosening in patients with osteoporosis, as it was shown to be able to suppress osteoblast apoptosis, promote osteogenic differentiation and improve bone quality around the prosthesis.

Wu et al., reported that the double-network crosslinked structures that formed between GelMA and liposomes significantly improved the hydrogel’s mechanical properties (Figure 3C,D) [101]. The inclusion of liposomes in the GelMA matrix in their study presented a sustained controlled release of the anticancer drug Gemcitabine for 4 days, whereas the free drug was released from a pure GelMA hydrogel in only 6 h. The loaded liposome-GelMA hydrogel killed MG63 cells in vitro and inhibited osteosarcoma in vivo, presenting itself as a promising implant for the treatment of osteosarcoma. In the field of wound healing, Kadri et al., reported that the nanofunctionalization of IPN GelMA-alginate hydrogels with rapeseed-derived liposomes significantly improved their mechanical properties and induced keratinocyte growth [104]. In another study, Yu et al., developed a liposome-GelMA hydrogel delivery system that controlled the release of the pro-healing chemokine stromal cell derived factor-1α, which might be used for clinical wound healing applications [103].

Chitosan is mainly composed of deacetylated (β-1,4-linked glucosamine) and acetylated (*N*-acetyl-d-glucosoamine) units with different degrees of deacetylation (70–95%) and molecular weights (10–1000 kDa) [117]. Chitosan’s low toxicity, biocompatibility, and biodegradability has led to its widespread use in hydrogels for tissue engineering and drug delivery applications [118]. Chitosan is also positively charged, which gives it antibacterial properties. Chitosan-based formulations exhibit good mucoadhesive characteristics and are capable of achieving a prolonged presence in the intestines and improving drug bioavailability in the GI tract. Although chitosan can be a very promising hydrogel for drug delivery applications, it has a limited capacity for controlling drug release. To overcome this disadvantage, liposomes and other nanovesicles can be embedded in the chitosan matrix to deliver drugs at a controlled rate.

Peers et al., studied the release of a model water-soluble dye (carboxyfluorescein), an antibiotic (rifampicin), and an anesthetic (lidocaine) from liposome-chitosan hydrogels [100]. The water-soluble molecules were first encapsulated in Dipalmitoylphosphatidylcholine (DPPC) liposomes, then embedded into chitosan physical hydrogels. This incorporation did not modify the hydrogel’s rheological properties. The release was sustained for longer periods in small unilamellar vesicles embedded in a chitosan hydrogel, compared to multilamellar vesicles embedded in a chitosan hydrogel and chitosan hydrogels without liposomes. Indeed, the liposome-chitosan hydrogel proved to be a promising candidate for the depot drug delivery of water-soluble antibiotics and anesthetics, which might have biomedical applications such as wound dressings.

Li et al., encapsulated curcumin inside liposomes and coated them with thiolated chitosan to form injectable and in situ-formable liposomal hydrogels [119]. The thermosensitive liposome-chitosan hydrogels could quickly transform from a fluidic state at room temperature to a gelled state at 37 °C. The release of curcumin was effectively delayed by the liposomal hydrogel encapsulation, which could improve the hydrogel’s water solubility and bioavailability in vivo. The cytocompatible liposome-chitosan hydrogels were able to suppress and kill MCF-7 breast cancer cells when loaded with curcumin. In summary, the injectable, in situ-formable, and thermosensitive liposome-chitosan hydrogels show great promise as scaffolds for the controlled drug delivery of curcumin or other anticancer drugs for breast cancer treatment or after tumor resection.

Fibrin is a blood coagulation product in vivo in the presence of thrombin enzymes, which catalyze the cleavage of fibrinogen to fibrin [120]. Fibrin is especially effective due to its unique properties, such as biodegradability and nontoxicity. In addition, fibrin’s components can be easily modified, such as the gel’s structure, mechanical properties, and degradation [121]. Wang et al., found that fibrin could be combined with liposomes and chitosan hydrogels to carry hydrophilic drugs with low-molecular weights [120]. This is especially important because fibrin, in addition to liposomes, can allow for a depot delivery system that controls the release of biologically active peptides or hydrophilic drugs. The gradual release of bioactive components can be achieved when using fibrin and liposome technology [122]. As for liposome-based hydrogels using alginate, they have been used for slow drug release as well as highly increased efficacy when compared to polymeric-based systems or liposome-based systems only [123,124].

### 5.2. Exosome-Loaded Hydrogels

Unlike liposomes, exosomes embedded in hydrogels are mostly used as bioactive molecules rather than as nanovesicles for the controlled delivery of drugs and molecules. The controlled release of exosomes from hydrogel systems increases their therapeutic efficiency by creating a depot of exosomes in the injury area, thus reducing the speed of their clearance from the body. Exosomes embedded in HA, gelatin, chitosan, and polypeptide-based hydrogels have been used for cartilage and bone defect repair, wound healing, and ischemia treatment, to name a few [16,18,109,110].

**Figure 4:**
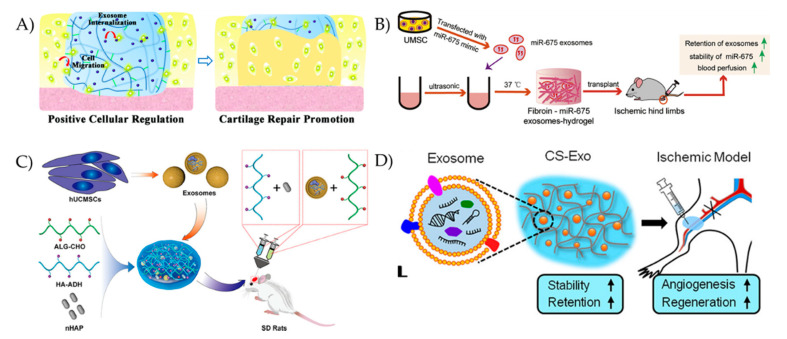
(**A**) Schematic illustration of the exosome-hydrogel scaffold for cartilage regeneration. Reproduced with permission from [109], Royal Society of Chemistry, 2017. (**B**) Schematic illustration of the miR-675-loaded exosome-silk fibroin hydrogel system for age-induced vascular dysfunction treatment. Reproduced from [112], Elsevier, 2019. (**C**) Schematic illustration of the exosome-hyaluronic acid-alginate hydrogel system for bone regeneration. Reproduced with permission from [110], American Chemical Society, 2020. (**D**) Schematic illustration of the exosome-chitosan hydrogel system for muscle regeneration. Reproduced with permission from [18], American Chemical Society, 2018.

Liu et al., embedded stem cell-derived exosomes in a photoinduced imine crosslinked hydrogel formed from the reaction of aldehyde groups generated under light irradiation of o-nitrobenzyl alcohol moieties modified HA and amino groups distributed on gelatin (Figure 4A) [109]. The exosome-hydrogel patch showed retained exosomes at defect sites and successfully integrated with native cartilage. It showed also good biocompatibility and remarkable operability, which suggests that it can be used as a scaffold for cartilage defect repair. In another study, to maintain stable exosomes at the deficient area and to repair bone degeneration in rats in vivo, Yang et al., successfully embedded stem cell derived exosomes in an injectable, hydroxyapatite-embedded, in situ crosslinked HA-alginate composite hydrogel system (Figure 4C) [110]. Their exosome-hydrogel system could significantly enhance bone regeneration.

Other than repairing cartilage, exosome-hydrogel systems can be used to repair chronic wounds. Wang et al., demonstrated this by producing a multifunctional, self-healing, injectable, and antibacterial polypeptide-based hydrogel that can control the release of embedded exosomes to treat chronic wounds [16]. This exosome-hydrogel system significantly increased the cellular proliferation, migration, and vascularization in vitro and significantly improved the wound healing of diabetic full-thickness cutaneous wounds in vivo. The exosome-hydrogel system also decreased the scar tissue area while inducing the appearance of abundant skin appendages which accelerated the diabetic wound healing process. This suggests that the controlled release of exosomes from the hydrogel had a synergistic wound healing ability.

Hindlimb ischemia treatment is another area in which exosome-hydrogel systems can be applied. Zhang et al., incorporated MSC-derived exosomes in a chitosan hydrogel matrix, which was injectable and could retain exosomes at the injury sites (Figure 4D) [18]. One of the main findings of their study was that the exosome-chitosan hydrogel promoted the therapeutic effects of exosomes, which led to an improvement in endothelial cells’ survival and angiogenesis, and an accelerated ischemic hindlimbs recovery. This exosome-chitosan system may be considered as a potential cell-free ischemia therapy. Han et al., demonstrated that miR-675, which is an aging process modulator, can be loaded in exosomes, that, in turn, can be embedded in a silk fibroin hydrogel to provide a sustained in vitro release and treat aging-induced vascular dysfunction (Figure 4B) [112].

Lv et al., revealed that exosomes incorporated in an alginate hydrogel were more efficient at stimulating angiogenesis, inhibiting cardiac apoptosis and fibrosis, while improving scar thickness and cardiac function when compared to only MSC-derived exosomes [113]. Shafei et al., loaded adipose-derived stem cell exosomes in an alginate-based hydrogel and concluded that this bioactive scaffold wound dressing technique induced collagen synthesis, wound closure, and tube formation in the wounded tissue [19].

A controlled-release of exosomes from synovium MSC was combined with chitosan and was observed by Tao et al., to stimulate human dermal fibroblast viability and proliferation. Furthermore, in a diabetic rat model, they found that this system improved the re-epithelialization stage of wound healing, activated vessel formation, and improved the collagen production in vivo [114]. In addition, Shi et al., studied exosomes from gingival MSC combined with a chitosan/silk hydrogel and their effects on a diabetic rat skin defect model, and found that this hydrogel could increase the wound healing of diabetic skin defects [115].

### 5.3. Hybrid Nanovesicle Releasing Hydrogels

To the best of our knowledge, no groups have examined the applications of hybrid exosome-liposome particles embedded in natural or synthetic hydrogels in vitro or in vivo yet. The only studies that have been done up until now using these hybrid particles, were only using free-standing nanovesicles [7,8,9,10]. Embedding theses hybrid particles in hydrogels is a very pertinent topic to investigate, since, as mentioned before, it can maximize the advantages of the targeting ability of exosomes and the versatility of liposomes while increasing the presence of these smart particles at the desired site, thus increasing their efficiency and the controlled release of bioactive compounds. Furthermore, building programmable release platforms is achievable using responsive hydrogels that can be chemically-, biologically-, electrically-, photo-, thermo-, or pH-responsive [125,126]. Coupling smart nanovesicles (hybrid exosome-liposome particles) with smart hydrogel systems (stimuli-responsive hydrogels) can create “smarter” delivery systems that can have big impact on drug and gene delivery, tissue engineering, and regenerative medicine fields.

## 6. Advantages of Hydrogel Systems for Efficient Drug Delivery

**Figure 5:**
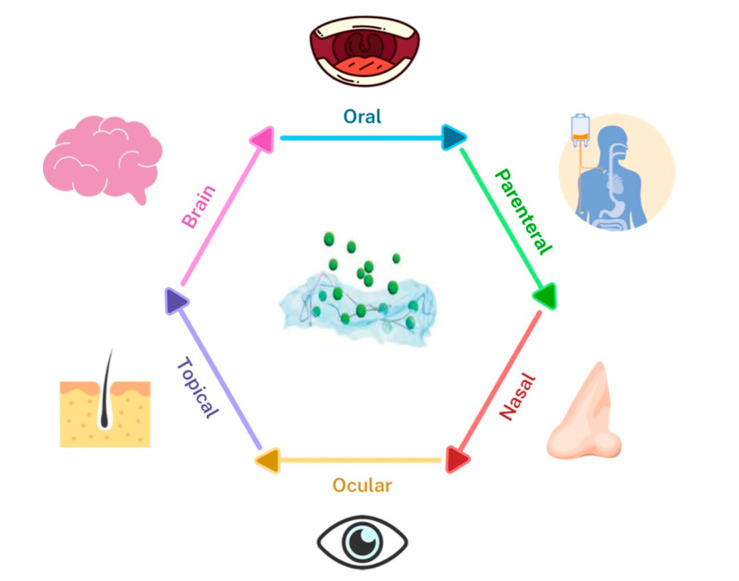
Schematic representation of the routes of administration of nanovesicle embedded hydrogel-based delivery platforms.

Despite all their advantages, such as targeting ability, controlled release of bioactive molecules and drugs, and biocompatibility, liposomes, exosomes, and hybrid particles are limited in their administration route, since they can only be administered via injection. Moreover, when they are injected in the body, these nanovesicles are quickly cleared from blood circulation and accumulate rapidly in the liver, spleen, lungs, and gastrointestinal tract. These challenges and limitations led to a shift from encapsulating and delivering drugs in nanovesicles only to embedding these loaded delivery nanosystems in hydrogels. When suspended in the hydrogel matrix, the controlled release period is extended from hours to days and even weeks, and the drug or nanovesicle delivery can be achieved via several administration routes and not only via injection, such as oral, nasal, parenteral, ocular, topical, and brain delivery (Figure 5).

Oral drug delivery is among the most common forms of drug delivery due to its ease and positive patient compliance. Gastroretentive drug dosage forms are favorable in order to prolong the gastric residence time so that bioavailability and therapeutic effects are improved. Oral routes are also favored due to the ability to protect the drug from enzymatic degradation [127]. Gutowska et al., focused on a new hydrogel delivery method that can exhibit delayed, zero-order, or on-off release profiles. The controlled delivery of the drug can assist with problems such as drugs decomposing too quickly in the stomach, or irritated stomach leading to adverse effects in the upper GI tract [128,129].

The parenteral route seems to be the favored route of administration for many drugs such as peptides and proteins. Hydrogels can be created to prolong drug release and gradually release the bioactive components to the patient. In addition, hydrogels can also increase drug half-life, increase bioavailability, protect drugs from enzymatic degradation, and decrease the frequency of drug administration, which could then lead to increased patient compliance [130]. Another positive component for some injectable hydrogels, such as chitosan, is that they are usually fluid at room temperature and viscous at body temperature. This gelation allows for sustained drug release and improved bioavailability.

The nasal route of delivery is typically used to treat certain ailments such as nasal allergies, congestion, and infections. However, recently, this route has been used for the delivery of small molecular weight polar drugs, proteins and peptides, in order to provide rapid uptake of the drug, something other routes fail to achieve [131]. Illum et al., reported in her paper that the most important limiting factor in the nasal route of drug delivery is the low membrane permeability. Another barrier that exists is the short nasal residence due to the mucosal turnover. Additionally, chitosan hydrogels have been known to be effective for nasal delivery due to their mucoadhesive, viscoelastic, and biocompatible properties. In turn, chitosan hydrogels can increase nasal residence time. Developments in the delivery route from nose to brain, and in maximizing rapid and highly concentrated drugs in the brain to elicit an efficient therapeutic response, are promising. Wu et al., studied a thermosensitive hydrogel and its prospective use for nasal drug delivery. The solution, when applied to the nasal cavity, turned into a viscous hydrogel at body temperature, reducing the rate of nasal mucociliary clearance and causing the drug to slowly release. Furthermore, Wu et al., explored quaternized chitosan as an absorption enhancer, leading to the capacity to open tight junctions between epithelial cells. They found that the hydrogel decreased the concentration of blood glucose (40–50% of the initial concentration) for 4–5 h post-administration, with no signs of cellular toxicity after application [132].

The ocular route has been met with some resistance in the field of drug delivery due to anatomical and physiological barriers that protect the eye from toxicants, though there are multiple ways to deliver drugs via the ocular route. These include topical, intravitreal, intracameral, and subtenon, among others. The benefits that follow include patient compliance, direct delivery to vitreous and retina, sustaining drug levels, and ease of administration. Some challenges that exist include higher tear dilution and turnover rate, toxicity due to high dosage, and cataracts, among others [133]. Gulsen et al., suggests that the mainstream route of eye-drops is ineffective, as 95% of the drug contained in the drops is lost due to tear drainage or absorption by the conjunctiva. Gulsen and coworkers proposed to encapsulate the drug in nanoparticles and to place them on the lens material. These contact lenses would ultimately release and deliver drugs over a long period of time [134]. Especially in treating ocular diseases and issues, a non-invasive delivery method, a maintained drug release, safety, and a high efficiency of drug encapsulation are desired. Thus, Kang Derwent and Mieler designed a sustained-release localized drug delivery system that was able to control the release of anti-VEGF agents to combat ocular vascular disease [135]. The developed hydrogel had thermoresponsive characteristics, so once the liquid was injected to the juxtascleral region via a small-gauge needle, the solution became a solid gel that released the encapsulated protein or anti-VEGF agent. Kang Derwent and Mieler argued that this system optimized the antiangiogenic effects and minimized the potential ectotopic effects of a large bolus delivery. They concluded that thermosensitized hydrogels had the ability to deliver drugs to the posterior segment of the eye in a steady, controlled fashion [135]. In Liu et al., they came up with an alginate hydrogel that supported human corneal epithelial cell growth using BSA as a drug model. Studies have shown that a composite hydrogel has the mechanical strength and optical clarity for use as a therapeutic lens and/or a corneal substitute for transplantation in corneal damage or diseases [136].

Topical, or transdermal drug delivery has been one of the more favored routes of drug delivery in recent years. There are three types of transdermal delivery systems: first-generation, second-generation, and third-generation. The first-generation delivery systems provide the delivery of lipophilic, small sized and low-dose drugs, while the second generation delivery systems use chemical boosters, ultrasound and iontophoresis that do not depend on cavitation. Finally, third-generation delivery systems use microneedles, thermal ablation, microdermabrasion, electroporation, and cavitation ultrasound to target the stratum corneum [137]. Overall, the topical route allows scientists to address the issue of low bioavailability and difficulties that arise from other routes of delivery. Targeting the stratum corneum while specifically protecting deeper tissues is a milestone that makes the topical route poised to make a widespread impact. In Calixto et al., they studied the effects of polyacrylic polymer hydrogels for topical use. They found that the polymer concentration raised the elastic, mechanical and bioadhesive characteristics of the hydrogel. Additionally, in an in vitro drug release test, they found that hydrogels controlled the release of the drug, improving the therapy outcome. They concluded that the polymeric hydrogels were promising platforms for bioadhesive topical drug delivery systems for the treatment of skin diseases [138]. In Reimer et al., they created a povidone-iondine (PVP-I) liposome hydrogel that allowed for both moist and antiseptic treatment and studied its effects [139]. In addition to the antimicrobial properties of PVP-I, it has been concluded that liposomes provided specificity to the target area, the ability to retain moisture, drug retardation, and prevented infections while activating the wound healing process.

Drug delivery via the brain is a difficult route due to the blood-brain barrier and the challenges it presents. Drugs, antibiotics, and neuropeptides all cannot overcome the barrier. However, nanoparticles seem to have the possibility to achieve desired therapeutic effects [140]. Nanoparticles have the potential to treat very aggressive brain tumors, among other things. The most likely mechanism would be through endocytosis by entering the endothelial cells of the brain blood capillaries [140]. Wang and co-workers also noted that the use of a hydrogel released in the subventricular zone to stimulate repair after a stroke decreased the stroke cavity size, increased neurons in the peri-infarct region and migratory neuroblasts, and decreased apoptosis [141].

## 7. Conclusions and Future Perspective

Since its discovery in 1965, liposome technology has massively advanced in terms of versatility. Liposomes have been extensively studied as drug delivery nanovesicles due to their ability to delivery bioactive molecules of different sizes and to target specific cells/tissues through the chemical modifications of their surfaces. On the other hand, surface chemical modifications are not required to create targeting exosomes, as they naturally possess this ability due to cellular and lipid adhesion molecules expressed on their surface. However, challenges in loading large bioactive molecules efficiently in exosomes have called for the development of a novel hybrid system based on the membrane fusion between liposomes and exosomes. This novel system has so far seen applications in cancer and gene editing and possesses great potential to be applied for many targeted drug delivery applications.

Many challenges related to liposomes and exosomes still persist. Without any doubt, liposomes are considered the most successful family within the field of nanomedicine. However, after 60 years of research, the full potential of the liposomes has yet to be fulfilled, as only a handful of liposomal drug formulations have reached the market. The main causes behind the low transition rate of liposomes from bench to bedside are their potential cytotoxic effects, leakage, stability problems, batch to batch reproducibility, effective sterilization methods, and scale-up problems. For exosomes, the field is still in its infancy, as clinical trials have just begun, and many challenges still need to be answered, such as inefficient drug loading, variable compositions and complex structures, possible safety issues, and the lack of optimized purification methods needed for large-scale production. A more comprehensive review about the challenges that the clinical translation of nanoparticles faces was written and recently updated by Anselmo and Mitragotri [142,143].

It has become evident that hydrogels have substantial potential to be used for pharmaceutical applications. There exist many challenges and hurdles that need to be surpassed before clinically approving a hydrogel product. These challenges were recently discussed in detail in a comprehensive review by Mandal et al. [144]. Nevertheless, in recent years, the FDA has approved a number of marketed hydrogel-based products such as Belotero balance^®^, Revanesse^®^ Versa^TM^, SpaceOAR^®^, Teosyal^®^ RHA, Radiesse^®^, and TraceIT^®^ [144,145]. Depending on the added drugs and bioactive compounds, hydrogels can be classified Class I, II, or III medical devices by the FDA [146]. A bright future stands ahead for commercialized hydrogel products, as the demand for patient-specific healing processes and treatments continues to grow by the day. Whether natural or synthetic, diffusion controlled or stimuli-responsive, a number of hydrogels have been developed for controlled drug delivery, each presenting a set of advantages and limitations. One approach used to limit the disadvantages of preferred natural hydrogels is nanofunctionalization with soft and hard nanoparticles. Nanofunctionalization with targeting nanovesicles can, in addition to ameliorating the mechanical properties of polymers, deliver drugs to one cell type in a certain tissue, which can be useful in reprograming and transdifferentiation applications.

Going forward, engineering effective targeted controlled drug delivery systems is of major importance and can achieve a huge breakthrough in treating many diseases, especially for cancer. These systems can form a depot around the tumor area, releasing smart nanovesicles encapsulating anticancer drugs in a controlled manner. This will lead to an increase in drug concentration in the tumor environment and to the targeting of cancer cells, while preserving healthy cells. In this review, we showed that hybrid exosome-liposome nanovesicles are great candidates for targeted drug delivery. However, because only a couple of groups have investigated such systems, more time is needed before we can fully judge the ability of this hybrid system. No research has been done yet on coupling this hybrid system with natural, synthetic, or stimuli-responsive hydrogels. Although, previous investigations of exosomes or liposomes embedded in hydrogels are promising.

## Figures and Tables

**Table 1:** Comparison of liposomes, exosomes, and hybrid particles embedded in natural hydrogel delivery systems and their applications.

Hydrogel	Loaded Molecule	Release Duration	Cell Type	Application	Ref.
Liposomes					
Gelatin methacryloyl (GelMA)	Deferoxamine, bovine serum albumin, and paclitaxel	5, 11 and 35 days	MC3T3-E1 and HUVECs	Bone regeneration	[15]
GelMA	Gemcitabine	4 days	MG63 cells	Osteosarcoma treatment	[101]
GelMA	Melatonin	25 days	MC3T3-E1 cells	Osteoporosis treatment	[102]
GelMA	SDF-1α	7 days	MSCs	Wound healing	[103]
GelMA and alginate	-	-	Keratinocytes	Wound healing	[104]
Collagen, gelatin, and alginate	Moxifloxacin and dexamethasone	1 day	Ocular epithelial cells	Corneal wound healing	[105]
Chitosan and alginate	mRNA	14 days	Fibroblasts and dendritic cells	Vaccine delivery	[106]
Chitosan	Carboxyfluorescein, rifampicin, and lidocaine	5.5 h	-	Wound dressings	[100]
Chitosan	-	-	HaCaT and hASCs	Tissue engineering scaffolds	[107]
Chitosan	α-tocopherol	6 days	L929 cells and cardiomyocytes	Cardiac tissue engineering	[108]
Exosomes					
Hyaluronic acid and Gelatin	-	-	hBMSCs	Cartilage regeneration	[109]
Hyaluronic acid and alginate	-	14 days	MC3T3-E1	Bone regeneration	[110]
Oxidative hyaluronic acid and Poly-ε-L-lysine	-	21 days	HUVECs	Skin regeneration	[16]
Modified hyaluronic acid	-	21 days	EPCs	Myocardial preservation	[111]
Silk fibroin	miR-675	36 days	H9C2 cells	Vascular dysfunction treatment	[112]
Chitosan	-	1 day	HUVECs	Hindlimb ischemia treatment	[18]
Alginate	-	10 days	HUVECs	Myocardial infarction treatment	[113]
Alginate	-	7 days	HeLa cells	Wound healing	[19]
Chitosan	miR-126-3p	6 days	HMEC-1 and fibroblasts	Wound healing	[114]
Chitosan and silk	-	-	GMSCs	Wound healing	[115]
Hybrid					
-	-	-	HeLa cells	Drug delivery	[7]
-	GFP mRNA	-	HUVECs, MSCs, and MDCK cells	Drug delivery	[8]
-	CRISPR/Cas9	-	MSCs and HEK293FT cells	Gene editing	[9]
-	doxorubicin	2 days	4T1, K7M2, and NIH/3T3 cells	Tumor targeted drug delivery	[10]
